# Nanogels as imaging agents for modalities spanning the electromagnetic spectrum

**DOI:** 10.1039/c5mh00161g

**Published:** 2015-10-19

**Authors:** Minnie Chan, Adah Almutairi

**Affiliations:** a Department of Chemistry and Biochemistry , University of California , San Diego , La Jolla , CA 92093-0600 , USA; b Skaggs School of Pharmacy and Pharmaceutical Sciences , KACST-UCSD Center of Excellence in Nanomedicine , Laboratory of Bioresponsive Materials , University of California , 9500 Gilman Dr., 0600 , PSB 2270 , La Jolla , San Diego , CA 92093-0600 , USA . Email: aalmutairi@ucsd.edu ; Tel: +1 (858) 246 0871

## Abstract

In the past few decades, advances in imaging equipment and protocols have expanded the role of imaging in *in vivo* diagnosis and disease management, especially in cancer. Traditional imaging agents have rapid clearance and low specificity for disease detection. To improve accuracy in disease identification, localization and assessment, novel nanomaterials are frequently explored as imaging agents to achieve high detection specificity and sensitivity. A promising material for this purpose are hydrogel nanoparticles, whose high hydrophilicity, biocompatibility, and tunable size in the nanometer range make them ideal for imaging. These nanogels (10 to 200 nm) can circumvent uptake by the reticuloendothelial system, allowing longer circulation times than small molecules. In addition, their size/surface properties can be further tailored to optimize their pharmacokinetics for imaging of a particular disease. Herein, we provide a comprehensive review of nanogels as imaging agents in various modalities with sources of signal spanning the electromagnetic spectrum, including MRI, NIR, UV-vis, and PET. Many materials and formulation methods will be reviewed to highlight the versatility of nanogels as imaging agents.

## Abstract 2

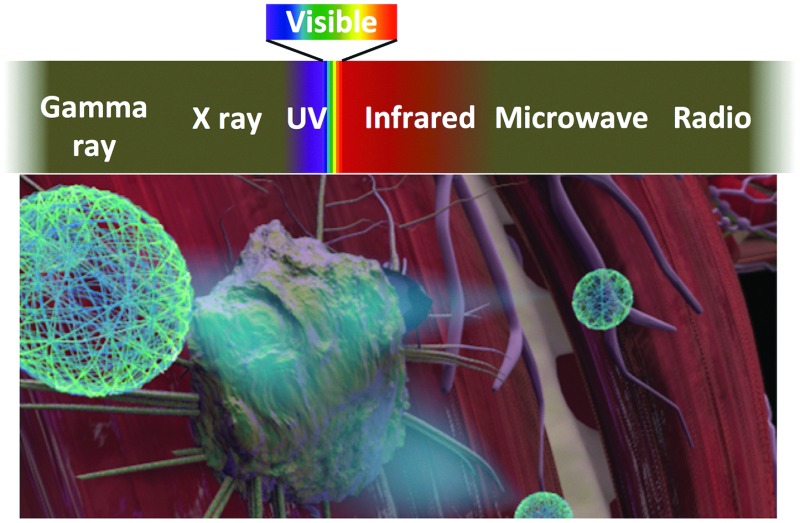
An updated and detailed overview of nanogel imaging agents for various modalities spanning the electromagnetic (EM) spectrum.

## 1. Introduction

Imaging is an essential part of clinical protocols that can provide morphological, structural, metabolic, functional and molecular information, as a minimally invasive procedure, for disease identification and assessment. Computed tomography (CT), positron emission tomography (PET), magnetic resonance imaging (MRI) and ultrasound are the most commonly used imaging modalities.^1,2^ To generate signal or enhance contrast, contrast agents are often used in the imaging regime. However, most clinically approved contrast agents are small molecules, which are rapidly cleared from the body, limiting the imaging window. In addition, these agents only provide overall whole-body contrast,^3,4^ and often lack disease specificity. An imaging agent that has optimal clearance, can be targeted to or respond to disease biomarkers would enable diagnosis and assessment with higher sensitivity, specificity, and efficiency.^3–5^

Nanomaterials, materials of submicron size, have opened up a new opportunity to overcome these challenges. Various studies have been performed on the development of micelles,^6–10^ polymeric nanoparticles,^11–13^ and dendrimers^14,15^ as imaging agents. The larger size of these macromolecular structures prevents their rapid clearance from the body through the renal system. The size and surface properties of nanoparticles can be modified to optimize their pharmacokinetics for imaging a specific disease. Increased imaging specificity would significantly improve accuracy in diagnosis, allowing better disease management planning and an improved prognosis. Additionally, nanoparticles have been demonstrated to improve the stability of encapsulated or attached probes.^16–19^

Hydrogel nanoparticles (nanogels) are hydrophilic nanosized polymeric networks that are held together in three dimensions through physical or chemical crosslinking.^20–23^ Nanogels have unique advantages over other nanoparticle imaging systems. They are highly biocompatible due to their high water content and consequent low interfacial tension with biological fluids, resulting in physical properties that resemble those of living tissues.^21–26^ Their size, ranging from 20 to 200 nm, can be controlled systematically through optimization of formulation parameters.^27^ In addition, nanogels can be designed to respond to environmental changes, such as temperature, pH, magnetic field, and ionic strength, with changes in their physiochemical properties, such as volume, water content, refractive index, interior network permeability, and hydrophilicity.^28^ This stimuli responsiveness can be exploited to design disease-responsive imaging nanogels. Nanogels also exhibit high loading efficiencies of both hydrophobic and hydrophilic molecules, including proteins, nucleic acids and quantum dots, and even act as chaperones to protect fragile molecules against degradation.^29–31^ Last but not least, release kinetics of nanogels can also be regulated by varying crosslinking density or incorporating stimulus-responsive crosslinkers. These properties make nanogels a promising platform for theranostic agents. Finally, nanogels possess high colloidal stability, essential for *in vivo* imaging/delivery agents.

**Fig. 1:**
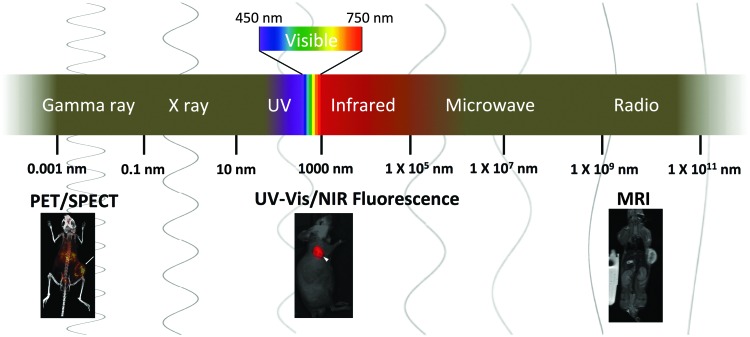
Imaging modalities with sources of radiation/signal spanning the electromagnetic spectrum.

Despite these advantages and the versatility of nanogels, there are limited reports of nanogel imaging agents. Two reviews on this topic have been published; however, they mainly focus on nanogels as drug carriers.^27,32^ Here, we provide an updated overview of nanogel imaging agents for various modalities spanning the electromagnetic (EM) spectrum (Fig. 1). Nanogel imaging agents will be categorized according to the wavelength of the imaging source: radiowave (MRI), near infrared (NIR), visible light, ultraviolet (UV), and gamma ray (PET) radiation (Fig. 1). Nanogel agents with imaging modalities other than the EM waves, such as ultrasound, have been reported but they are not discussed in this review.^33^ We will also provide an overview of the commonly used formulation methods, and discuss recent works on multimodal nanogel imaging agents, exploring how various materials and formulation methods can be employed to formulate nanogels for either *in vitro* or *in vivo* applications (Tables 1 to 4).

**Table 1:** (a) MRI contrast agents – part I. (b) MRI contrast agents – part II

Imaging modality	Dye/contrast agent	Means of incorporation	Polymer components	Responsive size change	Responsive imaging property	Formulation method^*a*^	Size (nm)	Targeting group	Cells/*in vivo* work	Therapeutics	Ref.
(a)											
MRI (Gd-based)	Gd-DOTA	As chemical crosslinkers	PAA chemically crosslinked by DTPA/DOTA crosslinkers	N/A	N/A	A	54–85	N/A	N/A	N/A	20
MRI (Gd-based)	Gd-DTPA	Conjugation	Poly(PEGMA)-crosslink by ethylene glycol bisacrylamide	N/A	N/A	B	10	N/A	Imaging of blood vessel in mice	N/A	54
MRI (Gd-based)	Gd-DTPA	Electrostatic	PAA + chitosan	pH (zeta potential)	N/A	A + D	220	N/A	Imaging of rabbit brain and liver	N/A	56
MRI (Gd-based) + NIR fluorescence	Gd^3+^ Cy 5.5	As physical crosslinkers (electrostatic) Conjugation	PEI	N/A	N/A	B	159 ± 62	N/A	Imaging of SCC7 tumor of mice	N/A	55
MRI (Fe_3_O_4_)	Fe_3_O_4_	Encapsulation	Dextrin-VA-SC16	N/A	N/A	C	100	N/A	Uptake in Murine bone marrow-derived macrophages	N/A	61
MRI (Fe_3_O_4_)	Fe_3_O_4_	As core from which polymer matrix was built on	Poly(PEGMA) crosslinked with MBA	N/A	N/A	A + E	68	N/A	N/A	Doxorubicin	63 and 64
MRI (Fe_3_O_4_)	Fe_3_O_4_	As core from which polymer matrix was built on	Poly(AEM·HCl) crosslinked with MBA	N/A	N/A	A + E	19	N/A	Healthy mice	N/A	65
MRI (Fe_3_O_4_)	Fe_3_O_4_	Encapsulation	Poly(NIPAAm-*co*-AA) crosslink by MBA	Temperature and pH	N/A	A + E	237 (pH 2.7) to 387 (7.6) 25 °C^*a*^	N/A	Cytotoxicity on HeLa cells	Doxorubicin	62
MRI (Fe_3_O_4_)	Fe_3_O_4_	Encapsulation	Self-assembly of poly(AAc-*co*-DSA); further coated by (poly(γ-GA-*co*-γ-GAOSu)-*g*-PEG-FA)	pH and magnetic hyperthermia	N/A	C + D	243 (pH 4.7) to 221 (pH 7.4) 20 °C^*a*^	Folate acid	Therapeutic efficacy on tumor-bearing mice	Doxorubicin	77

(b)											
MRI (Fe_3_O_4_) + UV fluorescence	Fe_3_O_4_ fluorescein	Encapsulation Conjugation	PBMA-*g*-(C12/fluorescein)	N/A	N/A	C	131–250	N/A	N/A	N/A	60
MRI (Fe_3_O_4_) + UV fluorescence	Fe_3_O_4_ fluorescein	Encapsulation Conjugation	pCBMA with disulfide crosslinks	Disulfide: reducing environment	N/A	A + E	110	RGD	Uptake in Macrophage cell	Fluorescently labeled dextran (model)	75
MRI (Fe_3_O_4_) + NIR fluorescence	Fe_3_O_4_ Cy5.5	Encapsulation Conjugation	Poly(NIPAAm-*co*-AA)	pH and/temperature	N/A	A + E	100 (pH 6.8) to 85 (pH 7.4) at 37 °C^*a*^	Lactoferrin	Imaging of C6 glioma in rats	N/A	66
MRI (Fe_3_O_4_) + UV fluorescence	Fe_3_O_4_ Dil	Encapsulation Encapsulation	Self-assembled poly(NIPAAm-*co*-AA) coated with PEI through electrostatic interaction	Temperature	N/A	C + D	200	N/A	Imaging of mice injected with nanogels-loaded hMSC	EPFG expression plasmid	68
MRI (Fe_3_O_4_) + UV fluorescence	MnFe_2_O_4_ fluorescent Schiff base bond (C=N) and double bond (C=C)	As core from which polymer graft on Part of polymer matrix	Self-assembled PLL crosslinked by poly-GA	N/A	N/A	B + E	<200	N/A	Imaging of mice injected with nanogels-loaded DC	N/A	69
MRI (19F)	19F-TFEMA	Part of polymer matrix	Poly-(DEAMA-*co*-TFEMA-EGDMA-PEG) crosslinker with ethylene glycol	pH	Signal is turned “On” as pH decrease	A	63 (pH 7.4) and 90 (pH 6.5)	N/A	N/A	N/A	72 and 73

^*a*^Formulation methods: refer to Fig. 1.

**Table 2:** PET imaging agents

Imaging modality	Dye/contrast agent	Means of incorporation	Polymer components	Responsive size change	Responsive imaging property	Formulation method^*a*^	Size (nm)	Targeting group	Cells/*in vivo* work	Therapeutics	Ref.
PET	^99m^Tc labeled 1-hexylcarbamoyl-5-fluorouracil (HCFU)	Encapsulation	Poly-(NIPAAM-*co*-VP) crosslinked by MBA; coated with h polysorbate 80	N/A	N/A	A	50	N/A	Biodistribution imaging of rabbits	HCFU	91
PET + UV fluorescence	^68^Ga-NODAGA Alexa Fluor 488	Conjugation Conjugation	Star-shaped PEG crosslinked by disulfide bond	Reducing environment	N/A	B	290	N/A	Uptake in monocytes	N/A	92
PET	^64^Cu-NOTA	As crosslinker	PAA chemically crosslinked with NOTA crosslinkers	N/A	N/A	A	63	N/A	Imaging of 4T1 tumor and metastasis of mice	N/A	93
PET	[18F]-labelled BoHc/A	Conjugation to encapsulated drugs	Amino group- and cholesteryl-group-bearing pullulan (cCHP)	N/A	N/A	C	40	N/A	Imaging delivery to nasal mucosa of mice	BoHc/A	84
PET + visible light	^68^Ga-DOTA NaYF4:Yb/Er/Tm UCNP	Conjugation As core from wit polymer was build on	PEI (coated on UCNP), conjugated with PEG	N/A	N/A	E	136	RGD	Imaging of M21 tumor bearing mice	N/A	94
PET + NIR	^64^ Cu-DOTA Cy5.5	Conjugation Conjugation	5β-Cholanic acid and azide groups modified glycol chitosan	N/A	NIR turned ON upon cleavage of MMP-sensitive peptide	C	∼300	MMP-sensitive peptide	Imaging of A549 tumor in mice	N/A	95
PET + NIR	^124^I-labeled chlorophyll-a cyanine dye	Encapsulation Conjugation	PAA	N/A	—	A	18–25	N/A	Imaging of Colon26 tumors in mice	Chlorophyll-a (PDT)	96

^*a*^Formulation methods: refer to Fig. 1.

**Table 3:** UV-vis imaging agents with QD or Au/Ag nanoparticles

Imaging modality	Dye/contrast agent	Means of incorporation	Polymer components	Responsive size change	Responsive imaging property	Formulation method^*a*^	Size (nm)	Targeting group	Cells/*in vivo* work	Therapeutics	Ref.
UV-vis	Fe_3_O_4_ QD	As core from which polymer was built on	Chitosan	pH	N/A	E	160	N/A	Uptake in L02 cells	Insulin	67
UV-vis	QD	Electrostatic interaction and chemical crosslinks with polymer matrix	CM-dextran and PLL	N/A	N/A	D + E	190	N/A	N/A	N/A	100
UV-vis	QD	Electrostatic interaction between HA and QD	HA	N/A	N/A	E	50–120	HA	Imaging of lymphatic vessels on ears of mice	N/A	99
UV-vis	QD	*In situ* synthesis inside the nanogels	Chitosan and PMAA crosslinked with MBA	pH	Photoluminescence (PL) increase as pH decreases	A + D	85–175	N/A	Uptake and cytoxicity on mouse melanoma B16F10 cells	Temozolomide	105
UV-vis	QD	Electrostatic interaction	Cholesterol-bearing amino-group-modified Pullulan	N/A	N/A	C + E	38	N/A	Uptake in HeLa cells	N/A	102
UV-vis	QD	Electrostatic interaction between lysosome, QD and polymer	Carboxymethyl cellulose	N/A	PL decreases as pH decreases	D + E	285	—	Uptake and cytotoxicity on HepG2 & MCF-7 cells	Methotrexate	101
NIR	QD	*In situ* synthesis inside the nanogels	HPC and PAA crosslinked wit MBA	pH and temperature	PL increases as pH decreases	A + D	32–50 (pH 4.5) to 75–83 (pH 7.4)	N/A	Cytotoxicity on B16F10 cells	Temozolomide	104
UV-vis	QD	As core from which polymer was built on	His-tagged polypeptides	pH, temperature and competitors	N/A	E	40.2	RGD	Uptake and cytotoxicity on HeLa cells	Dye ABDPSP & fluorescein sodium (model drugs)	103
UV-vis	Carbon shell and magnetic core nanoparticles	As core from which polymer was built on	Poly(NIPAM-AAm) crosslinked with MBA	NIR/magnetic induced thermal responsive	PL increases as temperature increase	A + E	320 (at 24 °C)	—	Cytotoxicity of B16F10 cells	Curcumin	106
UV-vis	Ag NP	As core from which polymer was built on	Poly(NIPAM-AA) crosslinked with MBA	pH	Blue shift and increase in absorption intensity as pH decreases	A + E	∼77 (pH 5.0) to ∼137 (pH 7.4) at 37 °C	—	Uptake and cytotoxicity on B16F10 cells	Dipyridamole	114
UV-vis	Au NP	As core from which polymer was built on	P(NIPAAM-*co*-AAm) crosslinked with MBA	Visible light induced thermal responsive	N/A	A + E	>80 (37 °C) and ∼53 (25 °C)	N/A	Uptake and cytotoxicity of HeLa cells	5-Fluorouracil	110
UV-vis	Au–Ag NP	As core from which polymer was built on	PEG crosslinked with PEGMA	NIR induced thermal responsive	PL increases as temperature increases	A + E	18–43 (37 °C)	HA	Uptake and cytotoxicity on B16F10 cells	Temozolomide	111
UV-vis	Au–Ag NP	As core from which polymer was built on	PS crosslinked with DVB and PEG crosslinked with PEGMA	NIR induced thermal responsive	N/A	A + E	22–37 (37 °C)	N/A	Uptake and cytotoxicity on B16F10 cells	Curcumin	112
UV-vis	Au NP	*In situ* synthesis inside the nanogel	Lysosome–dextran	N/A	N/A	C	190	N/A	Uptake and cytotoxicity on KB cells	Doxorubicin	114
UV-vis	Au NP	As core from which polymer was built on	Chitosan and PAA crosslinked with glutaldehyde	N/A	N/A	D + A	∼120	N/A	Uptake and cytotoxicity on HepG2 cells	N/A	113
UV-vis	Au NP	*In situ* synthesis inside the nanogel	Chitosan	N/A	N/A	B	80–230	N/A	—	N/A	115

^*a*^Formulation methods: refer to Fig. 1.

**Table 4:** UV-vis/NIR imaging agents with fluorescent dyes

Imaging modality	Dye/contrast agent	Means of incorporation	Major polymer backbone	Responsive size change/degradability	Responsive imaging property	Formulation method^*a*^	Size (nm)	Targeting group	Cells/*in vivo* work	Therapeutics	Ref.
UV-vis	5-Aminofluorescein (5-AF), 7-amino-4-methyl coumarin (AMC), or QDs	Conjugation	Chitosan and PAA crosslinked with MBA	N/A	N/A	A + D	180	N/A	*In vivo* lymphatic node mapping of Balb/c mice	Doxorubicin, or Vascular endothelial growth factor C	117
UV-vis	Fluorescein isothiocyanate	Conjugation	Poly(amidoamine) dendrimers and alginate crosslinked with Ca^2+^	pH	N/A	E + B	433	N/A	Uptake and cytotoxicity on CAL-72 cells	Doxorubicin	118
UV-vis	8-Hydroxypyrene-1-carbaldehyde (HPC)	Encapsulation	Polyurethane	—	Change in emission wavelength as a function of pH	B	92	N/A	Uptake in NIH/3T3 fibroblast cells	N/A	19
UV-vis/NIR	DMDP-M	Encapsulation	Crosslinked Pluronic F127	—	Fluorescence turned “on” in presence of thiols	B	45	N/A	Uptake in NIH/3T3 fibroblasts	N/A	123
UV-vis	Self-fluorescent abietane	As part of polymer matrix	Poly(abietane methacrylate) crosslinked with PEGDA	N/A	N/A	A	90–135	Folic acid	Uptake in MCF-7 cells	Doxorubicin	120
UV-vis	Self-fluorescent ALC linker	Electrostatic encapsulation	Branched PEI crosslinked by aldehyde-l-cystine (ALC) linker	Thiols	Decrease in pH and presence of reducing agents lowers the fluorescence	B	200	N/A	Transfection in multiple cell lines	pDNA	121
UV-vis	Rhodamine B	As part of polymer matrix	P(NIPAM-*co*-RhBUA) crosslinked with MBA	Temperature	Increased in fluorescence in presence of Cr^3+^ and increased temperature	A	117 (20 °C) and 50 (40 °C)	N/A	N/A	N/A	122
NIR fluorescence	Cyanine dye	Conjugation	Azide-functionalized PEG methyl ether crosslinked with l-cystine-*N*-carboxy anhydride	Reducing environment	N/A	B	210	N/A	N/A	Doxorubicin	127
	NIR-797 isothiocyanate	Conjugation	Methacrylated carboxymethyl cellulose crosslinked with disulfide linkage-containing crosslinkers	Reducing environment	N/A	B	192	N/A	NIR fluorescence imaging of H22 tumor-bearing mice	Doxorubicin	126
	IRDye800	Conjugation	Amine-cholesteryl-group-bearing pullulan	N/A	N/A	C	30	N/A	SLN Mapping in mice and pigs	N/A	130 and 131
	ICG	Conjugation	ICG–HA conjugate	HAdase	Fluorescence turn “On” in presence of HAdase	C	189	HA	Tumor and SLN imaging of MDA-MB-231 tumor bearing mice	N/A	134
	ICG	Conjugation	Self-assembled ICG–HA conjugate with surface further crosslinked with disulfide linkage-containing crosslinkers	HAdase	Fluorescence turn “On” in presence of HAdase	C + B	293	N/A	Imaging of front paw of mice that are injected with nanogels pre-incubated with HAdase	N/A	135
	ICG	Conjugation	HA + poly(beta-amino)ester (PBAE) + ICG	pH	Fluorescence turn “On” in presence of HAdase	D	73 (pH 7.4) and 15 (pH 5.5)	HA	Uptake in MDA-MB-231 breast cancer cells	N/A	136
	Ga-porphyrin	As crosslinkers	α,ω-Diazide/hydroxyl PEG and α,ω-difolate/azidePEG crosslinked by Ga-TPPPP	N/A	—	B	110	Folate	—	N/A	137

^*a*^Formulation methods: refer to Fig. 1.

## 2. Nanogels formulation methods

**Fig. 2:**
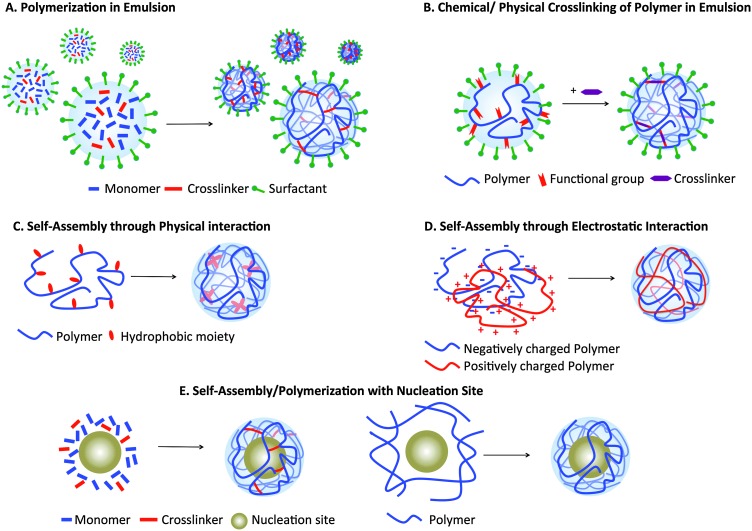
Nanogel formulation strategies. (A) Hydrophilic monomers and crosslinkers in a water-in-oil emulsion, stabilized by surfactants. Upon the addition of a catalyst, polymerization occurs within the emulsion droplets, forming nanogels. (B) Hydrophilic polymer modified with functional groups that allow physical/chemical crosslinking to form nanogels. (C) Polymer modified with hydrophobic moieties for self-assembly into nanogels. (D) Positively and negatively charged polymer self-assembly through electrostatic interaction. (E) Polymerization of monomers and crosslinkers shell or self-assembly of polymer modified with hydrophobic moieties in presence of nucleation sites.

Nanogels can be formulated using various materials and methods (Fig. 2). One of the most common is radical polymerization in an inverse emulsion^25,26,34,35^ (Fig. 2A), in which monomers, crosslinkers, and catalysts dispersed in aqueous droplets are stabilized in a continuous organic phase by surfactants; droplets become nanogels upon polymerization. Examples of commonly used monomers include acrylamide (AAm), pH-sensitive acrylic acids (AA) and thermoresponsive *N*-isopropylacrylamide (NIPAM). Inverse emulsion polymerization employs commercially available monomers and crosslinkers, can generate smart constructs if stimulus-responsive monomers and crosslinkers are incorporated, and allows control over mesh size by varying the crosslinking density. However, it requires surfactants that are difficult to completely remove and provides limited control over uniformity of nanogel size, often resulting in polydisperse particles.

Nanogels can also be formulated through crosslinking (either physical or chemical) of hydrophilic polymers that are modified with functional groups, such as thiols and acrylates^35–37^ (Fig. 2B), while amphiphilic polymer networks, such as cholesterol-modified pullulan, can self-assemble into nanogels (Fig. 2C). Although both methods require chemical modification of polymers, they allow formulation of nanogels using natural polymers, such as non-immunogenic polysaccharides (chitosan, hyaluronan, and dextran). The latter method does not require a surfactant, permitting a robust and facile formulation. However, since physical crosslinks are not stable, further chemical crosslinks are necessary for their *in vivo* application. Fig. 2D illustrates another formulation technique using a combination of oppositely charged polymer chains held together by electrostatic interactions. This approach does not require polymer modification, unlike the previous two methods, adding convenience and versatility. However, nanogels formed using this method are held together solely through physical interaction and so are not stable. Finally, nanogels can also be formed in the presence of nucleation sites, often inorganic nanoparticles such as iron oxide nanoparticles or quantum dots, on which the polymers or monomers are adsorbed and polymerization occurs (Fig. 2E). Nucleation sites work as a “template” on which nanogels are built, therefore generating nanogels of higher monodispersity. More details on each method of nanogel preparation have been published in other reviews.^23,38,39^

## 3. Magnetic resonance imaging (MRI)

MRI is one of the most frequently used clinical imaging techniques, employing a relatively low energy EM wave with wavelength ∼2.5 m (3 T) to 5 m (1.5 T). In addition to low ionizing radiation exposure, it also offers high anatomical resolution and great soft tissue contrast.^40,41^ Despite these advantages, MRI is limited by its low inherent contrast, a problem that can be overcome by contrast agents. Current clinically used MRI contrast agents can be divided into two categories: *T*_1_ and *T*_2_ contrast agents.^42,43^ *T*_1_ contrast agents are usually gadolinium (Gd) chelates, while *T*_2_ contrast agents are mainly iron oxide particles, including superparamagnetic iron oxide nanoparticle (SPIONs), ultra-small superparamagnetic iron oxide nanoparticles (uSPIONs) and very small superparamagnetic iron oxide nanoparticles (vSPIONs).

*T*_1_ agents enhance contrast by reducing the longitudinal relaxation time of the surrounding endogenous water, thus increasing the signal intensity and providing positive contrast. Conversely, *T*_2_ contrast agents provide negative contrast as they enhance water's transverse relaxation.^44^ The ability of an MRI contrast agent to provide contrast enhancement is characterized by its relaxivity, the measure of the change in the relaxation property of water per concentration of the contrast agent: 
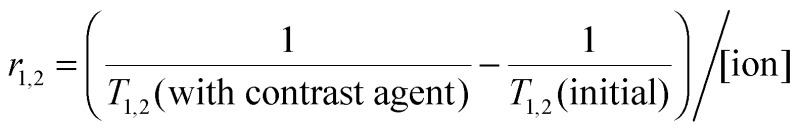
, where *r*_1_ corresponds to the longitudinal relaxivity, *r*_2_ corresponds to the transverse relaxivity, and [ion] corresponds to the concentration of the ions of the contrast agent.^44^

### 3.1 *T*_1_ agents

Most current clinical *T*_1_ contrast agents are small molecules, limiting relaxivity at the clinically relevant magnetic field of an MRI scanner (approximately 3 to 6 mM^–1^ s^–1^ at 1.5 T) due to their fast tumbling frequency.^42,45–47^ Thus, high-molecular-weight contrast agents,^45,47–49^ produced by encapsulation or conjugation of Gd-chelates with polymeric nanoparticles, dendrimers, micelles or polymers, that have higher relaxivities would be useful.^50–53^ Another advantage of nanogels for MRI is their high water content, allowing Gd ions to “relax” more water molecules in a given period than would be possible in more hydrophobic assemblies.

Currently, most strategies for the formulation of nanoparticles with Gd involve conjugation of Gd chelates to particles (Table 1a). Soleimani *et al.* conjugated Gd chelates on the surface of poly(ethylene glycol) methyl ether methacrylate nanogels crosslinked by ethylene glycol dimethacrylate.^54^ The nanogels, with a relaxivity of 17.5 mM^–1^ s^–1^ at 1.5 T, enhanced the overall signal intensity (blood vessels) in tumor-bearing mice substantially more than Magnevist at 20 minutes after injection. Yet, no tumor-specific signal enhancement or longer term imaging was shown to demonstrate the benefit of nanogels over small molecule contrast agents.

Without chelation, Lim *et al.* described the formulation of a nanogel MRI contrast agent through hybridization of poly(ethyleneimine) (PEI) with Gd^3+^ ions as the crosslinker.^55^ This design was intended to yield nanogels with increased elastic deformability to circumvent reticuloendothelial system (RES) sequestration and thus enhance tumor uptake. The nanogel surface was also modified with cyanine5.5 (Cy5.5) for NIR imaging. The 165 nm nanogels accumulated in SCC7 tumors in mice at a higher concentration than in the liver 12 h after injection. Unlike most other Gd-chelating particles, these nanogels exhibited a more significant transverse relaxivity (∼82.6 mM^–1^ s^–1^) than longitudinal relaxivity (2.1 mM^–1^ s^–1^), making them a better *T*_2_ contrast agent. *T*_2_-weighed MR imaging revealed negative contrast enhancement in the tumor 2 h after injection. Alternatively, Ahmed *et al.* recently published a nanogel system with chitosan and PAA as constituents.^56^ Negatively charged Gd–DTPA was adsorbed to the nanogels through electrostatic interaction with the positively charged chitosan. As the nanogels in these two papers carry Gd chelates or ions through physical interaction only, long-term Gd chelating stability, a critical safety concern for all clinical MRI contrast agents, may be an issue.

To address the concern of Gd-chelating instability, our group described a different formulation of a nanogel MRI contrast agent.^20^ Chan and Lux *et al.* synthesized three Gd-chelate crosslinkers that both held together polyacrylamide (PAAm) nanogels and stably retained Gd^3+^ ions. Incorporation of the Gd-chelating crosslinker into nanogels enhanced relaxivity by 4 to 6 times (18 mM^–1^ s^–1^ at 1.5 T) and essentially prevented transmetallation by Zn^2+^, the ions most likely to displace Gd^3+^ *in vivo*.

### 3.2 *T*_2_ agents

Most superparamagnetic contrast agents incorporate water-insoluble iron oxide crystals consisting of magnetite (Fe_3_O_4_) or maghemite (γ-Fe_2_O_3_) with a core diameter in the range of 4 to 180 nm.^57–59^ Iron oxide particles are of considerable interest as contrast agents because of their low toxicity. Due to their hydrophobicity, they are often encapsulated in nanoparticles to enhance their solubility in aqueous solution. In addition, the clustering of iron oxide particles inside nanoparticles allows them to work synergistically in enhancing *T*_2_ relaxation of water, leading to a higher relaxivity.

Multiple studies have employed similar strategy in formulating Fe_3_O_4_-encapsulated nanogels by utilizing Fe_3_O_4_ as a core to build the hydrogel layer (Table 1a and b). Amphiphilic polymers, such as poly(butyl methacrylate) (PBMA) grafted with 1-dodecylamine (C12)^60^ and hydrophobized dextrin (a carbohydrate polymer),^61^ can self-assemble into nanogels encapsulating Fe_3_O_4_ nanoparticles in the hydrophobic core. This strategy is convenient, as Fe_3_O_4_ templates the one-step assembly of nanoparticles through hydrophobic–hydrophobic interactions between Fe_3_O_4_ and the hydrophobic moieties of the polymer. Alternatively, emulsion polymerization of monomers and crosslinkers, which adsorb onto Fe_3_O_4_ through inverse emulsion, has also been used to formulate nanogels.^62^ For example, Sun *et al.* and Liu *et al.* employed a commonly used stealth material, PEG methacrylate, and *N*,*N*′-methylenebisacrylamide (MBA, also called bisacrylamide in this review) to form their Fe_3_O_4_-carrying nanogel system.^63,64^ Likewise, Gong *et al.* encapsulated Fe_3_O_4_ in amine-containing nanogels prepared by photopolymerizing *N*-(2-aminoethyl) methacrylamide hydrochloride monomers.^65^ In this method, Fe_3_O_4_ assists the formation of a nanogel shell: the high surface area of Fe_3_O_4_ allows adsorption of monomers and crosslinkers at high concentrations, while strong UV absorption of Fe_3_O_4_ initiates the formation of radicals for the photopolymerization. All of these Fe_3_O_4_-encapsulating nanogels ranged in size from 68 nm to 250 nm, with *T*_2_ relaxivities ranging from 100 to 440 mM^–1^ s^–1^. As shown with these examples, encapsulation of Fe_3_O_4_ inside nanogels offers a simple way to formulate *T*_2_ contrast agents with the flexibility of using various materials as coating. However, the clinical potential of a *T*_2_ agent is limited. They offer negative contrast, darkening areas in which they accumulate; this makes interpretation and identification of lesions more difficult than with a *T*_1_ agent. In addition, Fe_3_O_4_, such as Feridex® or Resovist®, are readily taken up by reticuloendothelial cells in the liver and spleen. This limits their use to only liver lesion imaging. Expansion of their applications would require substantial materials engineering and innovative designs.

### 3.3 Dual MR and fluorescence imaging agents

While MRI provides excellent soft tissue contrast, it suffers from low sensitivity. The combination of MRI with other imaging modalities, such as PET or fluorescence, which have higher sensitivities, can compensate for this disadvantage of MRI and provide better information about disease location and progression and therapeutic efficacy. Various groups have reported nanogels with dual MR and fluorescence imaging properties, often by encapsulating Fe_3_O_4_ into nanogels and conjugating (or encapsulating) fluorescent dyes/quantum dots.^67^

**Fig. 3:**
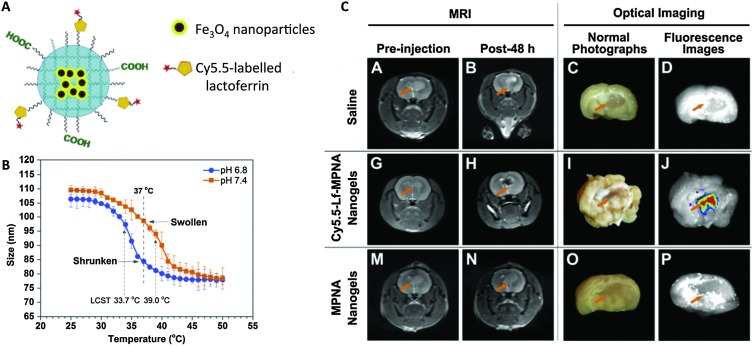
pH- and temperature-sensitive nanogels as dual *T*_2_ and optical imaging agents. (A) Cy5.5-Lf-MPNA: P(NIPAM-*co*-AA) nanogels encapsulating Fe_3_O_4_ nanoparticles and conjugated with Cy5.5-labeled lactoferrin as a glioma-targeting ligand. (B) Thermo- and pH-responsive change in nanogel size (due to change in hydrophobicity). (C) *In vivo* MR and *ex vivo* NIR fluorescence imaging showed higher uptake of Cy5.5-Lf-MPNA than MPNA (without Cy5.5-labeled lactoferrin) in rat glioma.^66^

Jiang *et al.* formulated a pH- and thermo-sensitive nanogel as a dual MRI and fluorescence agent for medical imaging of brain glioma (Fig. 3).^66^ Fe_3_O_4_ was encapsulated in p(NIPAM-*co*-AA) (PNA), and the resulting nanogels were further conjugated with Cy5.5-labeled lactoferrin for fluorescence and tumor targeting. The pH sensitivity of the polymer translated to a lowered lower critical solution temperature (LCST) at slightly acidic pH (6.4), which caused nanogels to shrink at physiological temperature, enhancing uptake into tumor cells. MRI imaging revealed that uptake of lactoferrin-labeled nanogels into brain glioma of rats 48 h after injection was significantly higher than that of unlabeled nanogels. Park *et al.* utilized the same polymer to formulate a theranostic nanogel for both MRI and fluorescence imaging to visualize gene delivery.^68^ p(NIPAM-*co*-AA) self-assembled into nanogels around the amine-functionalized Fe_3_O_4_. Instead of chemically conjugating a dye, Dil was encapsulated. The nanogels were further coated with polyethyleneimine (PEI), which carries positively charged amines, for complexation with negatively charged DNA for gene delivery. Nanogels complexed with green fluorescent protein (GFP) plasmid DNA were internalized in human mesenchymal stem cells (hMSCs), leading to GFP expression. The transplantation of these hMSCs into mice was then monitored using MRI and fluorescence. Of these two studies, only the second demonstrated imaging by both modalities *in vivo*, and neither compare the data obtained from the two modalities. Thus, they do not fully demonstrate how MRI and fluorescence complement each other in providing morphological and functional information.

Kim *et al.* employed a different approach for the development of dual MRI and fluorescent nanogels. Instead of encapsulating or conjugating a dye, these researchers used an autofluorescent nanogel matrix for tracking dendritic cell therapy.^69^ Positively charged poly(l-lysine) (PLL) was added to negatively charged poly(γ-glutamic acid) (γ-PGA)-coated manganese/iron oxide nanoparticles (MnFe_2_O_4_) to form ionic nanogels; PLL amines were crosslinked by the addition of glutaraldehyde, which was responsible for its fluorescence at around 553 nm. Incubation of dendritic cells with the nanogels allowed MR and fluorescence visualization of their accumulation in lymph nodes for dendritic cell therapy. It should be noted that fluorescence is very sensitive, making it well suited to complement the low sensitivity of MR imaging. However, its limited penetration depth in the UV to visible range may not be effective in imaging of tumors/lymph nodes deeper than a few millimeters inside the body.^70,71^ The systems covered in this section are good proofs-of-concept, showcasing the versatility of modifying nanogels as bimodal agents, yet likely have limited clinical utility.

### 3.4 Fluorine-19 (^19^F) MRI probes

In addition to Gd and iron oxide-based contrast agents, ^19^F-based agents have recently gained increasing attention. As the abundance of water in the body leads to a high background signal in ^1^H MRI, magnetic species that are less abundant allow a higher signal-to-noise ratio. One such species is ^19^F, whose signal has a similar sensitivity to that of ^1^H, and is relatively safe. Most ^19^F agents developed so far have been perfluorocarbon-based polymeric nanoparticles or micelles.

Oishi *et al.* translated this concept to nanogels by formulating a pH-activated ^19^F MRI nanogel system through co-polymerization of ^19^F-bearing 2,2,2-trifluoroethyl methacrylate (TFEMA), pH-sensitive 2-(*N*,*N*-diethylamino)ethyl methacrylate (DEAMA), and ethylene glycol dimethacrylate crosslinkers.^72,73^ At pH 7.4, nanogels were hydrophobic and shrunken due to deprotonation of amines, reducing molecular motion of ^19^F compounds in the hydrophobic gel core and thus broadening signal (off state). The signal was turned on at pH 6.5 as amines were protonated and nanogels became hydrophilic and swollen. Though ^19^F MR imaging was done only *in vitro*, the elegant design of the nanogels and high pH sensitivity of their system provides new insight into development of ^19^F-containing nanogels as tumor-responsive imaging agents.

### 3.5 MR theranostic imaging agents

Considerable effort has been made toward the development of MRI theranostic agents, which have both MR imaging and drug delivery capabilities, using micelles or polymeric nanoparticles.^74^ Characteristic properties of diseases, for example, low extracellular pH, hypoxia, and the reducing environment of tumors, can be exploited in the design of disease-specific theranostic agents. However, reports of nanogel MRI theranostic systems are still scarce.

As a proof of concept Zhang *et al.* formulated a reduction-sensitive nanogel, with a disulfide-containing crosslinker, encapsulating Fe_3_O_4_ using non-fouling carboxybetaine methacrylate (CBMA) as a monomer.^75^ Dithiothreitol **(**DTT)-induced degradation of nanogels resulted in the release of model drugs (fluorescein isothiocyanate (FITC)-labeled dextran) and a reduction in relaxivity which translated to higher MR signal intensity. Since DTT is a harsh reducing agent, glutathione (GSH), an abundant thiol reducing agent that maintains the redox state in cells, would have allowed a more biologically relevant proof of principle; this species is upregulated in many diseases, including tumors.^76^ while the study shows the potential of nanogels as reduction-responsive theranostics, high sensitivity to the low GSH concentrations found *in vivo* (millimolar range) remains a major challenge.

Chiang *et al.*, on the other hand, utilized another property of tumors, low extracellular pH, to develop a tumor-specific theranostic system. Fe_3_O_4_ and Dox were encapsulated into self-assembled pH-sensitive poly(acrylic acid-*co*-distearin acrylate) polymersomes, stabilized by an electrostatically assembled nanogel shell of positively charged chitosan and a negatively charged folic acid (FA)-tagged block copolymer for active targeting.^77^ Similar to the other nanogel theranostic study, *in vivo* imaging and drug delivery was not evaluated. Though achieving sensitive disease-responsive MR imaging with optimal release kinetics for drug delivery remains a challenge, the studies mentioned here suggest how stimuli-responsive materials together with proper system design would allow potential realization of nanogels as MRI theranostic agents.

### 3.6 CEST agents

In addition to the previously mentioned MRI contrast agent chemical exchange saturation transfer (CEST) agents have gained substantial interest in the last decades.^78,79^ CEST offer multiple advantages over other conventional contrast agents as it provides frequency-dependent signal, therefore signal can be switched “off” and “on”. This unique property also allows potential visualization of multiple imaging agents and quantitative characterization of *in vivo* environment with ratiometric method. Both diamagnetic and paramagnetic CEST agents have been incorporated into nanocarriers, specifically liposomes, to increase the number of exchangeable protons for contrast sensitivity enhancement.^80–82^ Though there are currently no report on nanogels-based CEST agents, the capability of nanogels to encapsulate large amount of molecules and their high water content make nanogels an attractive platform CEST agents.

## 4. PET imaging

In addition to MRI, positron emission tomography (PET) is also commonly used for clinical molecular imaging, especially in oncology, neurology and cardiology. PET uses gamma rays, which have the highest energy among imaging modalities (wavelength in order of 10^–12^ m) compared to MRI, its sensitivity is very high, requiring only nanomolar or even lower concentrations of the imaging agent,^1,83,84^ allowing visualization and quantification of disease markers. PET signal arises from correlated gamma photons generated upon annihilation of electrons by tracer-emitted positrons. These photons are generated some distance away from the site of emission, as annihilation occurs when positrons lose enough kinetic energy to collide with an electron.^85,86^ Commonly used tracers in organic molecules include ^18^F and ^11^C. Among these, ^18^F fluorodeoxyglucose, a glucose analog, is often used as a tracer for oncological imaging because many tumors consume more glucose than surrounding tissues.^87^ Other studies mainly focus on metal radionuclei with longer half-lives, such as copper-64 (^64^Cu), yttrium-86 (^86^Y), and zirconium-89 (^89^Zr).^88,89^ gallium-68 (^68^Ga) has gained increasing attention since the development of modern ^68^Ge/^68^Ga generators, as their generation does not require an on-site cyclotron.^90^

### 4.1 PET nanogels

Although many polymers, micelles and polymeric nanoparticles have been used in experimental macromolecular radiopharmaceutics, the use of nanogels as PET imaging tracers has rarely been explored (Table 2). Soni *et al.* published the first radiolabeled nanogels in 2006, encapsulating *N*-hexylcarbamoyl-5-fluorouracil (HCFU), a prodrug of 5-fluorouracil (FU), into nanogels for drug delivery to the brain.^91^ A ^68^Ga-labeled polysorbate 80 coating granted the nanogels with gamma imaging properties. Instead of using a coating, Singh *et al.* labeled the nanogel matrix directly by attaching ^68^Ga-chelating 1,4,7-triazacyclononane-1-glutaric acid-4,7-diacetic acid (NODAGA) to the arms of a hydroxy- and thiol-terminated star-shaped poly(ethylene oxide-*stat*-polypropylene oxide) prepolymer.^92^ Nanogels were formed by self-assembly, followed by crosslinking of thiols on the polymer, which were sensitive to reducing environments. Although radiochemical yield was measured *in vitro* in a competition experiment, the *in vivo* stability of the system should be further investigated.

**Fig. 4:**
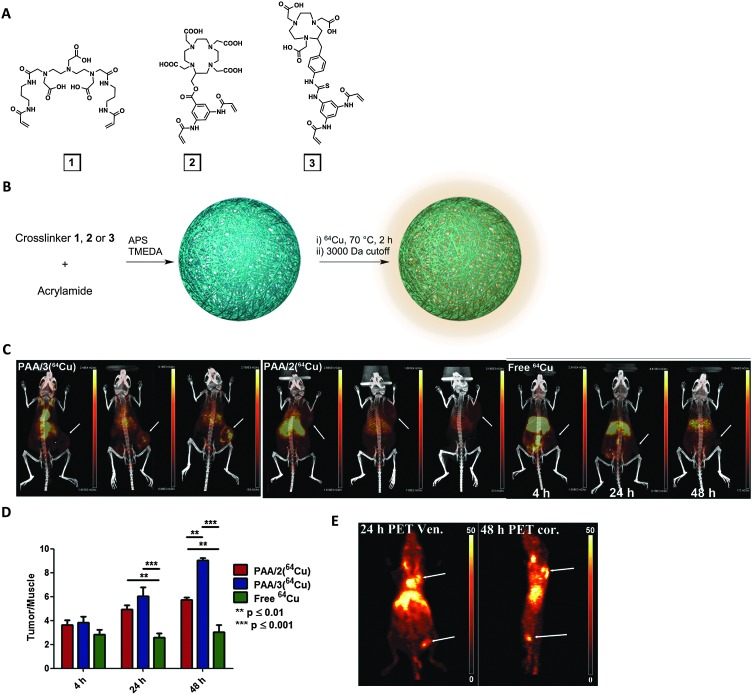
^64^Cu-bearing polyacrylamide (PAAm) nanogels as PET imaging nanogels. (A) Structures of three metal-chelating crosslinkers: DTPA (1), DOTA (2) and NOTA (3)-based. (B) Acrylamide was used as monomer and either 1, 2, or 3 was used as crosslinker to formulate nanogels (PAA/1, PAA/2 or PAA/3). (C) PET-CT imaging of mice with 4T1 tumors at 4 h, 24 h and 48 h after injection with PAA/2, PAA/3 or free ^64^Cu^2+^. Arrows indicate tumors. (D) Tumor/muscle ratio of PET signal. (E) PET imaging (arrows indicate popliteal lymph node in leg and primary tumor on shoulder) at 24 or 48 h post-injection.^93^

Recently, Lux *et al.* altered the chemistry of the metal-chelating crosslinkers used in our MRI nanogel contrast agent to formulate a PET nanogel tracer (Fig. 4).^93^ ^64^Cu was selected because of its advantageous long half-life (12.7 h). To enhance Cu-chelating stability, a 1,4,7-triazacyclononane-1,4,7-triacetic acid (NOTA) crosslinker was synthesized and used in the PAAm nanogel formulation, allowing retention of 94% of the copper in the nanogels after 48 h of incubation in serum. *In vivo* PET imaging revealed high nanogel uptake in 4T1 mouse breast tumors. The tumor/muscle intensity ratio was greater than nine at 48 h after injection, which clearly delineated the tumor. In addition to primary tumors, the nanogels were also able to image metastasis, highlighting this system's clinical potential as a tumor-imaging PET agent. While PET has superior sensitivity for functional imaging, MRI offers unmatched soft tissue resolution for anatomical imaging. Combined PET and MRI therefore provides information about both structure and function, which can significantly improve disease identification, localization and thus the diagnostic evaluation. This new imaging field also opens up the opportunity to develop bimodal PET imaging agents, particularly after the commercialization of fully-integrated whole-body PET–MRI scanner. Simultaneous PET and MRI scanning can not only shorten imaging time, but also reduce motion artifacts. The above ^64^Cu-chelating and Gd-chelating crosslinkers could thus be combined in one nanogel system for simultaneous PET and MRI.

### 4.2 Dual PET imaging

Other groups have developed bimodal PET nanogel imaging agents. Gallo *et al.* developed a dual PET/fluorescence imaging nanogel, in which fluorescent upconverting nanoparticles, NaYF4:Yb/Er/Tm (18 : 1.5 : 0.5 mol%), were encapsulated in a PEI shell.^94^ The amines on PEI allowed conjugation with ^68^Ga-chelating 1,4,7,10-tetraazacyclododecane-1,4,7,10-tetraacetic acid (DOTA) and iRGD, a tumor-cell-targeting peptide. The 136 nm nanogels absorbed NIR light and emitted visible light; whole-body PET imaging of mice with M21 melanoma tumors allowed quantification of nanogels in various organs. Uptake of iRGD-labeled nanogels in tumors was approximately 30% greater than that of unlabeled nanogels. Both fluorescence and PET imaging offer great sensitivity for functional imaging but have low three-dimensional spatial resolution. Therefore, MRI or CT would be better choices for coregistration with PET to provide complementary anatomical and morphological information.

Recently, Lee *et al.* published a dual PET and metalloprotease-activable optical imaging probe prepared from glycol chitosan nanogels (CNP).^95^ Glycol chitosan modified with 5β-cholanic acid and azide groups self-assembled into nanogels *via* hydrophobic interactions among the 5β-cholanic acid moieties. Using bio-orthogonal click chemistry with surface azides, ^64^Cu-chelating DOTA and an activable matrix metalloproteinase (MMP)-specific peptide probe, bearing Cy5.5 NIR dye and black hole quencher 3 (BHQ3) on opposite ends, were conjugated onto the nanogels' surfaces. MMP is overexpressed in many tumors and is responsible for tumor progression and metastasis. Quenched fluorescence was recovered only upon peptide cleavage by the target MMP. While the always “on” PET imaging signal can overcome the shortcomings of fluorescence imaging, such as limited tissue penetration depth, MMP-triggered “off-to-on” NIR fluorescence provides tumor-imaging specificity, which would improve tumor diagnosis accuracy.

Gupta *et al.* employed a different approach, instead loading PAA nanogels with ^124^I-labeled porphyrin and conjugating fluorescent cyanine dyes to surface amines to prepare dual PET-NIR fluorescence theranostic agents.^96^ The inclusion of porphyrin confers possible theranostic applications as a photodynamic therapy agent. In contrast to the previous system, in which the radioisotopes are chemically conjugated to nanogels, this system may suffer from leakage of encapsulated ^124^I-labeled porphyrin.

As mentioned above, PET is an essential tool in clinical imaging given its superior sensitivity. However, this modality alone does not provide any structural and morphological information. Combination with CT or MRI is required to identify where the PET signal originates in the body. In particular, MRI, because it does not use strong ionizing radiation, is highly preferable to CT. This encouraged the recent rapid development of novel and effective dual PET–MRI imaging agents for simultaneous PET and MR imaging with a new level of precision and resolution.

## 5. UV-visible/near-infra-red optical imaging

The popularity of optical imaging as an *in vitro* analytical tool is driven by its ease of use and high sensitivity (similar to PET) without ionizing radiation.^97,98^ These advantageous properties also enable their use in imaged-guide surgeries.^2^ Optical fluorescence can be categorized into three ranges according to the emission wavelength: near-infra-red (NIR) from 750 nm to 1000 nm, visible light from 450 nm to 750 nm and ultraviolet from 320 nm to 450 nm. In the last decade, advancements in hardware, imaging probes designs and mathematical models have made *in vivo* whole-body fluorescence imaging possible. This imaging modality is especially attractive given its sensitivity, allowing for the detection of fluorescent dyes in the picomolar range. In addition, quantum dots, gold/silver nanoparticles, and fluorescent dyes with various excitation and emission wavelengths, photostabilities, quantum yields, and functional groups are commercially available. Delivering these fluorescent entities using nanogels can improve their solubility, *in vivo* stability, and pharmacokinetics.

### 5.1 Quantum dots

Quantum dots (QDs), usually nanocrystals of the semiconductor cadmium selenide (CdSe), have attracted attention as imaging probes due to their unique properties: high resistance to photobleaching, high quantum yield, narrow emission peak, and commercial availability at various emission wavelengths ranging from UV to NIR.

**Fig. 5:**
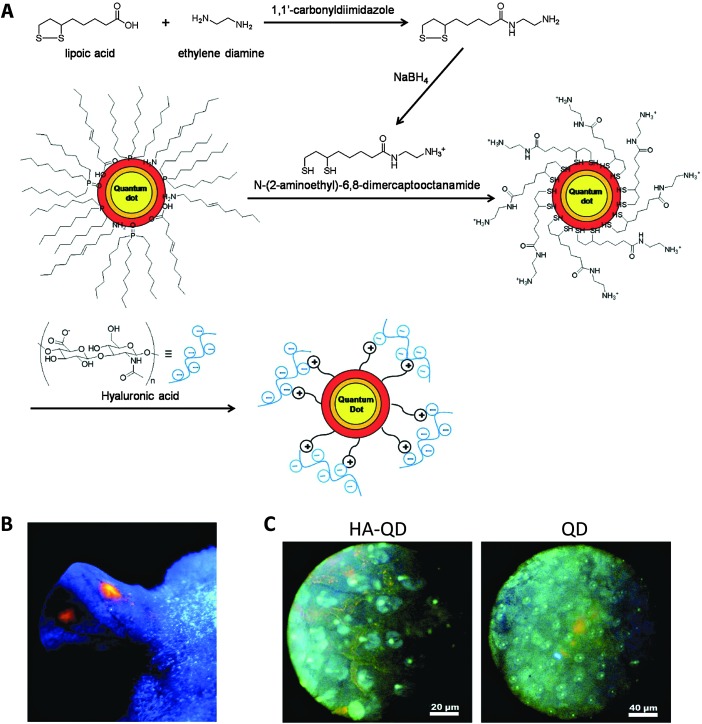
QD-encapsulating nanogels as lymph node imaging agents. (A) Synthesis and formulation scheme of HA-QD nanogels. (B) Image of a mouse ear under UV lamp 30 minutes after subcutaneous injection of HA-QD. (C) Fluorescence images of lymphatic vessels under microscope 30 minuets after injection of either HA-QD or QD. HA-QD provides a clear delineation of lymphatic vessel.^99^

Instead of being loaded into nanogels like other small dyes or drugs, QD can act as a core to template nanogels formation (Table 3). Surface ligand exchange of QD allows modification of QD with surface charges to electrostatically interact with oppositely charged polymers or monomers that form the nanogel matrix.^99–102^ The physical attraction between the QD and polymer matrix can minimize unwanted QD release. For example, QD was modified with a surface ligand carrying thiol groups at one end for QD surface adsorption, and amine groups on the other end for positive charges (Fig. 5).^99^ The positively charged QD electrostatically attracted the negatively charged hyaluronic acid (HA) polymer during formation of nanogels. Since HA has been shown to bind to lymphatic vessel endothelial receptor 1 (LYVE-1), this group employed the fluorescent HA nanogels to visualize lymphatic vessels, which may be used in lymphangiogenesis imaging for better understanding of cancer progression. Subcutaneous injection of the nanogels into mouse ears showed their accumulation in lymphatic vessels with strong fluorescence signal for up to a day. Yang *et al.* did not rely on electrostatic interactions, instead relying on the affinity between CdSe–ZnS QD and polyhistidine tag to build a nanogel for imaging cellular drug delivery.^103^ The polypeptide also included two hydrophobic and one hydrophilic domain to form a sandwiched layer for encapsulating both hydrophobic and hydrophilic drugs.

Another strategy to formulate QD nanogels is through *in situ* synthesis of QD.^104,105^ Wu *et al.* utilized chitosan interpenetrated with polymethacrylic acid (PMAA) as the nanogel matrix.^105^ Chitosan's abundant hydroxyl groups sequestered Cd^3+^ and stabilized CdSe QDs formed *in situ* inside the nanogels; chemical crosslinking and hydrophobic interactions enhanced stability. Chitosan's amines (–NH_2_) and carboxylates (COO^–^) from PMAA gave the nanogels a pH-dependent volume phase transition. At approximately pH 5, the electrostatic interactions between deprotonated carboxylates (COO^–^) and protonated amines (NH_3_^+^) caused maximal shrinking; as pH increased (>pH 5.5) amines were deprotonated decreasing interactions with COO^–^ and causing repulsion among polymers, swelling the nanogels. Not only did this enhance release of the encapsulated anticancer drug, temozolomide (TMZ), but changes in the protonation state of the counterions COO^–^ and NH^3+^ also caused changes in the local dielectric environment of the QDs. This yielded pH-dependent optical properties: quenched NIR photoluminescence became unquenched as pH increased. This study demonstrated the application of nanogels as NIR theranostic agents for tumor drug delivery and pH sensing.

Carbon dots, with even higher biocompatibility and lower toxicity, have recently been investigated as an alternative to QD. Wang *et al.* encapsulated magnetic iron oxide nanocrystal cores and carbon dots in a carbon shell, which were further encapsulated into a poly(NIPAM-*co*-AA) nanogel.^106^ The temperature-dependent emission of carbon dots at 377 nm allowed sensing of the environmental temperature. In addition, NIR irradiation of the carbon shell or application of an alternating magnetic field induced localized heating and triggered drug release.

While QD-encapsulated nanogels have the advantage of direct and easy fabrication, the high toxicity of QD associated with the release of free Cd^3+^ remains a problem. In addition, though QD are widely commercially available at various emission wavelengths, their absorption lies in UV-blue ranges, which further limits their application as *in vivo* agents.

### 5.2 Au/Ag nanoparticles or nanorods

Gold (Au) or silver (Ag) nanoparticles with sizes larger than 3 nm exhibit strong surface plasmon resonance (SPR) absorption in the visible spectrum.^107,108^ The SPR originates from the oscillation of conductive electrons on the surface upon irradiation at resonant wavelengths. The surface plasmon band depends on various factors, including nanoparticle size, shape, and the surrounding environment. These metallic nanoparticles have the advantage of being less susceptible to photobleaching, and with absorption and emission orders of magnitude greater than those of small fluorescent dyes, making them better suited for imaging. Finally, because these nanoparticles can generate heat by absorbing NIR light, they can also provide photothermal treatment, and be formulated as theranostic agents.

Similar to QD, surface modified Au/Ag nanoparticles can act as a core for a hydrogel shell to be built on,^109–113^ or could be synthesized *in situ* within nanogels (Table 3).^114,115^ Wu *et al.* developed multiple thermo or pH responsive nanogels with metallic nanoparticle cores for cellular imaging, chemo- and NIR-induced photothermal therapy. In one study, they encapsulated bimetallic Ag–Au nanoparticles into thermo-responsive PEG-based nanogels. In addition to fluorescence imaging, Ag–Au nanoparticles allowed conversion of NIR light to heat for photothermal therapy. Thermo-responsive PEG polymeric shells switched from hydrophilic to hydrophobic as the temperature increased, shrinking the nanogels, increasing photoluminescence, and releasing the hydrophilic model anticancer drug (temozolomide). The temperature responsiveness could be tuned by changing the thickness of the polymer shell. HA has been shown to bind CD44 receptors, which are overexpressed in various cancers and contribute to cell proliferation and migration,^116,117^ The HA conjugated-PEG nanogels enabled uptake by CD44-overexpressing B16F10 murine melanoma cells through endocytosis; these cells emitted fluorescence when excited at 405 nm. The therapeutic efficacy, as measured by cytotoxicity, of the combined chemo and photothermal therapy was higher than the additive effect of either alone. The group subsequently improved the system to include a hydrophobic polystyrene gel layer inside the PEG shell to encapsulate hydrophobic drugs.^112^ Their studies demonstrated the advantage of Ag/Au nanoparticles over other fluorescent dyes or QD, as they can provide photothermal therapy in addition to optical imaging, which allows them to be developed into multifunctional theranostics.

### 5.3 Small fluorescent dyes

A wide range of fluorescent dyes in the UV-vis to NIR range have been synthesized and studied as they are frequently used in microscopy. Conjugation or encapsulation of small dyes to nanogels provides a simple and direct route to formulate fluorescent nanogel imaging probes (Table 4). One strategy is to conjugate dyes to the nanogel surface post-formulation.^118,119^ Fluorescent dyes can also be modified as monomers or crosslinkers to be used in nanogels formulation.^120–122^ Incorporation of dyes in the particle matrix can minimize spontaneous release of labels due to instability *in vivo*, which complicates image interpretation,^120^ and avoids the need for excessive washing to remove dyes adsorbed to nanogels. Chen *et al.* utilized this strategy and modified autofluorescent abietane-based acid as a methacrylic monomer which was crosslinked with PEG diacrylate to yield a nanogel for both drug delivery and imaging. FA was conjugated to the surface as a targeting ligand, and doxorubicin was loaded and stabilized by the hydrophobic abietane. Fluorescent imaging showed uptake into MCF-7 human breast cancer cell cytoplasm within 1 h, illustrating their potential as cellular drug delivery tracking agents. Encapsulation of dyes into nanogels without covalent attachment has also been reported.^19,123^ Cao *et al.* encapsulated a pH-sensitive dye, 8-hydroxypyrene-1-carbaldehyde (HPC), into polyurethane nanogels as an intracellular fluorescence pH indicator.^19^ The nanogels' pH sensitivity allowed imaging of H_2_O_2_-induced cytosolic acidosis.

While the small size and resulting high cellular uptake make UV-visible fluorescent nanogels good for sensing and detecting intracellular states, their use as *in vivo* imaging agents is limited. As shown in the above examples, almost all of these optical nanogels system were examined as cell imaging agents only. This choice may relate to the overlap of these nanogels' absorption and emission with the absorption of hemoglobin, melanin and other proteins in the body (from 200 to 650 nm),^70,98,124,125^ and interference by autofluorescence from tissues in this wavelength range.

To achieve deep tissue penetration and accurate detection, research on optical imaging agents has focused on those with absorption and emission wavelengths in the NIR region (from 750 nm to 1000 nm), in which absorption and autofluorescence from biological tissue is substantially lower. Generally, NIR probes can be categorized as either inorganic, such as quantum dots, gold (Au) nanorods/nanoclusters/nanoparticles and upconverting phosphor (UCP), or organic, such as cyanine, squaraine, BODIPY® and Alexa Fluor dyes® *etc.* ^124^ These probes have been thoroughly compared elsewhere.^124^ Most of them suffer from low water solubility, a drawback for *in vivo* applications. In addition, the rapid degradation of organic NIR dyes *in vivo* prevents their wide use in clinical applications. Encapsulation or conjugation to hydrophilic nanogels can enhance their stability in aqueous solution, making them suitable for *in vivo* imaging and potentially enabling NIR imaging in humans.

#### 5.3.1 NIR imaging of drug delivery

**Fig. 6:**
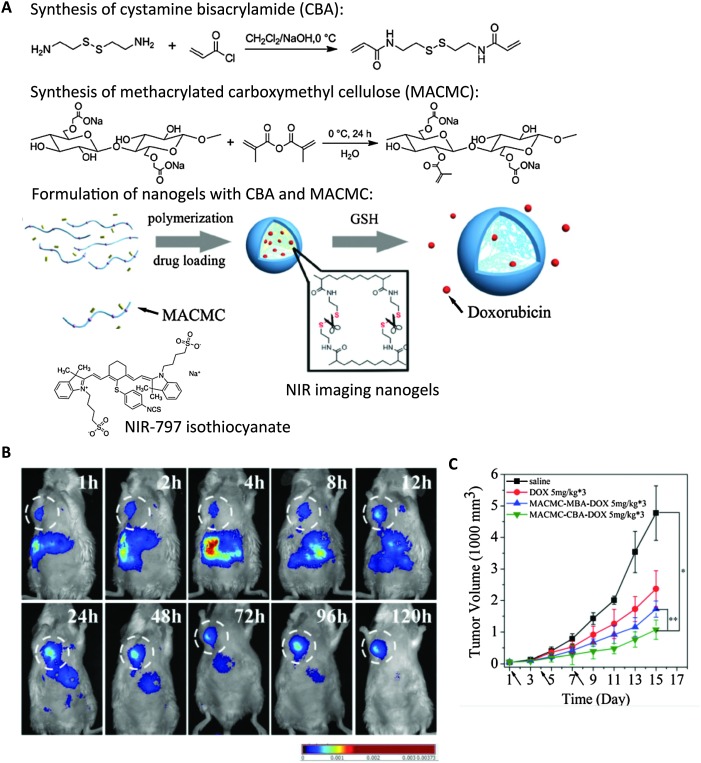
Cellulose-based nanogels for NIR tumor imaging. (A) Synthesis of disulfide crosslinkers (CBA), methacrylated cellulose (MACMC) and formulation of nanogels encapsulating doxorubicin (Dox) through radical polymerization. NIR-797 isothiocyanate was conjugated to render nanogels with NIR emitting properties. (B) Real-time NIR imaging of subcutaneous hepatic H22 tumor-bearing mice injected with NIR nanogels at 120 h post-injection. White circle highlights the tumor. (C) Tumor size was substantially decreased upon multiple dosage of Dox-containing nanogels compared with controls. (dosage time indicated by arrows). Saline, free Dox, and nanogels formulated with bisacrylamide (MACMC–MBA–Dox) without reduction-sensitive drug release were injected as controls.^126^

Attachment of NIR dyes to drug-delivery nanogels agents allows their *in vivo* tracking. Xing *et al.* and Qian *et al.* both formulated reduction-sensitive NIR fluorescent drug delivery nanogels with disulfide bond-containing crosslinkers, and conjugated cyanine dye or IR-797 isothiocyanate respectively.^126,127^ In Qian's study, they demonstrated that the accumulation and distribution of nanogels in H22 hepatocellular carcinoma tumor-bearing mice through intravenous injection could be clearly visualized through real-time whole-body NIR fluorescence imaging (Fig. 6).^126^ This study clearly illustrated the advantageous *in vivo* application of NIR probes.

#### 5.3.2 NIR lymph node imaging agent

Other than imaging drug delivery, NIR nanogels have also been used to identify the sentinel lymph node (SLN), the first lymph node from which tumor cells spread.^128,129^ Particularly in breast cancers, SLN identification followed by removal for biopsy is important for metastasis assessment. While the SLN is often located by intratumoral injection of methylene blue or radio-isotopes,^128,129^ small dyes lack SLN specificity and often diffuse into regional lymph nodes. Noh *et al.* improved the pharmacokinetics of SLN imaging agents by conjugating IRDye800, to cholesterol-modified pullulan nanogels of 30 nm,^130^ enhancing dye photostability. More importantly, NIR imaging of mice injected intradermally with the NIR nanogels in their front paws revealed that, at 30 min post-injection, the signal intensity in the SLN was six times higher than in those injected with free dyes. Experiments performed on larger animals such as pigs and dogs using a similar system (IRDye900-conjugated pullulan-cholesterol nanogels) also showed that they are better at identifying SLN than free dyes.^131^ These results show that NIR nanogels have great promise to be developed as clinical imaging probes for N-staging, as they have longer retention in regional lymph, allowing a more flexible imaging regime with less auxiliary diffusion than small dyes.

### 5.4 NIR tumor imaging

NIR nanogels have also been examined as tumor imaging agents. The enzyme hyaluronidase (HAdase), which is responsible for the degradation of hyaluronic acid (HA), is upregulated in tumor cells and contributes to tumor progression, angiogenesis, and metastasis.^132,133^ Overexpression of HAdase can thus serve as a biomarker for cancer progression. Mok *et al.* designed a hyaluronidase-activable NIR tumor-imaging nanogel using indocyanine green (ICG) dye-conjugated HA.^134,135^ ICG-conjugated HA self-assembled into nanogels through the hydrophobic interaction among ICG dyes. Park *et al.* utilized another tumor biomarker, low extracellular pH, for tumor-specific imaging in their system with ICG dyes.^136^ ICG dyes were encapsulated inside the nanogels composed of HA polymer and pH-sensitive poly(beta-amino)ester (PBAE) through electrostatic and hydrophobic interaction. In all three cases, embedding the dyes inside nanogels quenched the NIR signal; presence of HAdase or low pH liberated ICG and turned on the NIR signal, allowing tumor-specific imaging. Mok *et al.* showed that their nanogel system allowed longitudinal NIR imaging of tumors for up to three days post-injection. Instead of conjugation to a dye, Fu *et al.* formulated label-free NIR nanogels by incorporating a Ga-porphyrin complex as a crosslinker.^137^ Ga-porphyrin was modified with tetra-alkyne functionalities and reacted with difunctionalized azide-PEG through Cu^2+^-catalyzed click chemistry, to form nanogels of 30 or 120 nm. Though these nanogels exhibit maximum emission at approximately 725 nm, *in vivo* imaging was not shown to illustrate their feasibility as *in vivo* NIR tumor imaging agents.

## Conclusion

In this review, we discussed nanogels as imaging agents in various imaging modalities spanning the electromagnetic spectrum. Imaging agents for each imaging modality have different requirements. They need to be able to carry molecules at different concentrations, of different sizes, and different chemical properties, ranging from small fluorescent/NIR dyes, metal-chelates, quantum dots to iron oxide nanoparticles. Because of the availability of numerous formulation methods and building materials, nanogels can be utilized as imaging agents in many imaging modalities, making them very versatile. Specifically, due to flexibility in their constituents, more biocompatible and less immunogenic materials, such as natural polymers (chitosan, dextran, and pullulan), can be used. This ideal characteristic of nanogels gives them great potential as a platform for clinical contrast agents. However, the development of nanogels as imaging agents is in its infancy. As reviewed herein, while a few systems have been tested as *in vivo* whole body imaging/theranostic agents for imaging tumors, most have only been used *in vitro*. Increasing structural stability, *in vivo* contrast, photostability, and disease-specific accumulation, and reducing toxicity and overcoming background signal, still remain major challenges for nanogels' full realization as *in vivo* imaging agents. Despite these problems, fast advances in imaging hardware and the constant discovery and design of newer and safer materials suggest that these obstacles could be overcome in the near future for applications in oncological, neuropsychiatric and cardiac imaging.
